# Impact on survival of the number of lymph nodes resected in patients with lymph node-negative gastric cancer

**DOI:** 10.1186/s12957-015-0602-x

**Published:** 2015-06-01

**Authors:** Xiaoyuan Chu, Zhong-Fa Yang

**Affiliations:** Department of Medical Oncology, Jinling Hospital, School of Medicine, Nanjing University, Nanjing, Jiangsu Province 210000 China; Division of Hematology-Oncology, Department of Medicine, University of Massachusetts Medical School, Worcester, MA 01655 USA

## Abstract

**Background:**

Patients with lymph node-negative gastric cancer show a better overall survival rate than those who have a pathological lymph node-positive gastric cancer. But a large number of patients still develop recurrence. We aimed to explore the significant prognostic factors of lymph node-negative gastric cancer and determine how many lymph nodes should be removed.

**Methods:**

A total of 3103 patients who underwent radical operation are identified from the Surveillance, Epidemiology, and End Results database. Standard survival methods and restricted multivariable Cox regression models were applied.

**Results:**

The overall survival rate was significantly higher with an increasing number of negative lymph node resected. Among the 843 patients who had the exact T stage, the overall survival rate was significantly better in T3-4 group with more than 15 lymph nodes resected (*P* < 0.001) but not in T1-2 stage patients (*P* = 0.44). A further 25 more lymph nodes resection did not show additional survival benefits. Multivariate analysis of patients demonstrated that age, depth of tumor invasion, and the number of lymph nodes resected were the significant and independent prognostic factors.

**Conclusions:**

A lymphadenectomy with more than 15 lymph nodes removal should be performed for T3-4 lymph node-negative gastric cancer. But the survival benefit of a lymphadenectomy with more than 25 lymph nodes removal is disputed. And the further treatment should refer to the prognostic indicators.

## Background

Gastric cancer is the fourth common malignant tumor worldwide and the prognosis of this cancer remains poor [1]. The 5-year overall survival rate is approximate 25 % [2]. Nodal metastases in gastric cancer represent an important prognostic indicator after surgical resection, and it is widely recognized that patients who received a standardized pattern of lymph node dissection may get more survival benefits [3–5]. Although patients with lymph node-negative gastric cancer show a better overall survival rate than those who have a pathological lymph node-positive gastric cancer, recurrence occurred in a large number of patients. Furthermore, little is known about the prognostic factors in lymph node-negative gastric cancer after radical surgery [6].

Several reports demonstrated that the depth of tumor invasion is an independent prognostic factor of lymph node-negative gastric cancer. But there is still controversy on the prognostic significance of other factors, such as patient age, tumor size, and patterns of lymph node resection [6–9]. Meanwhile, the most recent edition (seventh) of the International Union against Cancer (UICC) and the American Joint Committee on Cancer (AJCC) do not define the necessary minimum number of lymph nodes for resection, especially for gastric cancer patient at stage pN0. In this work, we evaluated the prognostic factor of patients with lymph node-negative gastric cancer. We further explored the optimal number of lymph node resection for accurate staging and more survival benefits in the patients with lymph node-negative gastric cancer after radical dissection.

## Methods

### Patients

The Surveillance, Epidemiology, and End Results (SEER) program of the National Cancer Institute collected and published cancer incidence and survival data from population-based cancer registries. Data collected include patient demographic information, pathological characteristics, and survival data from 1973 to 2009. The exclusion criteria are (1) patients who did not have an exact pathological diagnosis; (2) gastric cancer which ICD-O-3 code without the range of 8000–8152, 8154–8231, 8243–8245, 8250–8576, 8940–8950, and 8980–8990; (3) patients who did not have an exact tumor size; (4) patients who did not have an exact pathological grade; (5) patients whose postoperative survival time was less than 3 months; (6) patients with tumor location at the esophagogastric junction (site code 160) were also excluded to be consistent with the seventh edition of the AJCC Cancer Staging Manual, which now stages these patients under esophageal scheme; (7) patients with distant metastasis; and (8) patients with no lymph node removed. After screening patients with the above criteria, we obtained a total of 3103 patient data sets with lymph node-negative gastric cancer for further retrospective analysis.

### Statistical methods

Patients with lymph node-negative gastric cancer were classified into lymph node-negative group 1 (LNN 1) to lymph node-negative group 4 (LNN 4) on the basis of the following criteria: LNN 1, up to three (0–3) lymph nodes removed; LNN 2, three to seven (3–7) lymph nodes removed; LNN 3, seven to fifteen (7–15) lymph nodes removed; LNN 4, more than fifteen lymph nodes removed. These cutoff points were chosen by stratifying patients into different groups with different numbers of lymph nodes removed. The median survival times for each LNN subgroup are as follows: 45 months for LNN 1, 50 months for LNN 2, 54 months for LNN 3, and 58 months for LNN 4. For tumor size, previous studies used 5 cm as the cutoff point; we divided the group with tumor size less than 5 cm into some more detailed sub group in order to make a more accurate analysis. For LNN groups, in Western countries and Eastern countries, different surgical methodologies are chosen. The cutoff points are chosen according to AJCC metastatic lymph node stages, we deem that a bit more lymph nodes resected is the precondition to make an accurate lymph node stage. Thus, four groups were established by combining the neighborhood survival curves using the log-rank test [10]. The exact T stage was determined according to the seventh edition of the AJCC Cancer Staging Manual and the corresponding SEER code.

Survival analysis and curves were calculated from observed postoperative survival time according to the Kaplan-Meier method and compared with the log-rank test. Multivariate analyses were calculated in terms of the Cox proportional hazard model. The chi-square test was used to evaluate the statistical significance of differences. Continuous data were presented as the mean ± standard deviation (SD). Statistical analyses were performed using SPSS 13.0 for windows. All statistical tests were conducted two-sided, and *P* values <0.05 were considered to be statistically significant.

## Results

### Patient characteristics

There were 3103 patients in the SEER database who fulfilled the critical selection criteria between 1973 and 2009. Demographic and pathologic characteristics of LNN 1 and LNN 4 were summarized in Table 1. In all patients, 37,715 lymph nodes were examined (median 9, mean 12.15, range 1–90). Median age was 73 years (range 23–98). Among the 3103 patients, 1760 (56.7 %) were males. One thousand eight hundred twenty of 3103 patients were white people, 446 were black, 19 were indigenous populations of the Western hemisphere, 618 were East Asians (including Chinese, Japanese and Korean), and the rest were 200 patients including Filipino, Hawaiian, Vietnamese, Laotian, Kampuchean, Thai, Asian Indian or Pakistani, Samoan and 3 unknown. The tumor size was divided into six subgroups, and most of them were less than 5 cm (*n* = 2197, 70.8 %). For the tumor depth statistics, only 843 of the total 3103 patients got an exact AJCC T stage (only patients who were diagnosed later than 2004 in the database have enough details to make an accurate T stage according to the AJCC seventh edition).

**Table 1:** Demographic and pathologic characteristics of LNN 1 to LNN 4

	LNN 1 (*n* = 582) *n* (%)	LNN 2 (*n* = 708) *n* (%)	LNN 3 (*n* = 938) *n* (%)	LNN 4 (*n* = 875) *n* (%)
Age (years)				
≤65	133 (22.9 %)	189 (26.7 %)	274 (29.2 %)	311 (35.5 %)
>65	449 (77.1 %)	519 (73.3 %)	664 (70.8 %)	564 (64.5 %)
Gender				
Male	311 (56.9 %)	419 (59.2 %)	531 (56.6 %)	479 (54.7 %)
Female	251 (43.1 %)	289 (40.8 %)	407 (43.4 %)	396 (45.3 %)
Race				
White	392 (67.4 %)	460 (65.0 %)	527 (56.2 %)	441 (50.4 %)
Black	86 (14.8 %)	116 (16.4 %)	136 (14.5 %)	108 (12.3 %)
Indigenous Westerner	6 (1.0 %)	5 (0.7 %)	4 (0.4 %)	4 (0.5 %)
East Asian	73 (12.5 %)	100 (14.1 %)	195 (20.8 %)	250 (28.6 %)
Others	25 (4.3 %)	27 (3.8 %)	76 (8.1 %)	72 (8.2 %)
Location				
Upper	20 (3.4 %)	28 (4.0 %)	43 (4.6 %)	39 (4.5 %)
Middle	50 (8.6 %)	67 (9.5 %)	102 (10.9 %)	126 (14.4 %)
Lower	241 (41.4 %)	290 (41.0 %)	407 (43.4 %)	298 (34.1 %)
Overlapping	271 (46.6 %)	323 (45.6 %)	386 (41.2 %)	412 (47.1 %)
Tumor size (cm)				
Diameter ≤1	67 (11.5 %)	65 (9.2 %)	82 (8.7 %)	89 (10.2 %)
1< Diameter ≤2	120 (20.6 %)	137 (19.4 %)	164 (17.5 %)	125 (14.3 %)
2< Diameter ≤3	95 (16.3 %)	122 (17.2 %)	146 (15.6 %)	139 (15.9 %)
3 <Diameter ≤4	94 (16.2 %)	107 (15.1 %)	167 (17.8 %)	121 (13.8 %)
4< Diameter ≤5	70 (12.0 %)	88 (12.4 %)	97 (10.3 %)	102 (11.7 %)
Diameter >5	136 (23.4 %)	189 (26.7 %)	282 (30.1 %)	299 (34.2 %)
Depth of invasion				
T0 and T1	54 (9.3 %)	71 (10.0 %)	123 (13.1 %)	129 (14.7 %)
T2	15 (2.6 %)	26 (3.7 %)	48 (5.1 %)	53 (6.1 %)
T3	24 (4.1 %)	43 (6.1 %)	78 (8.3 %)	84 (9.6 %)
T4	10 (1.7 %)	19 (2.7 %)	29 (3.1 %)	37 (4.2 %)
Unknown	479 (82.3 %)	549 (77.5 %)	660 (70.4 %)	572 (65.4 %)
Grade				
Well differentiated	59 (10.1 %)	74 (10.5 %)	80 (8.5 %)	77 (8.8 %)
Moderately differentiated	217 (37.3 %)	250 (35.3 %)	346 (36.9 %)	265 (30.3 %)
Poorly differentiated	294 (50.5 %)	360 (50.8 %)	490 (52.2 %)	507 (57.9 %)
Undifferentiation	12 (2.1 %)	24 (3.4 %)	22 (2.3 %)	26 (3.0 %)

### Overall survival analysis

**Fig. 1:**
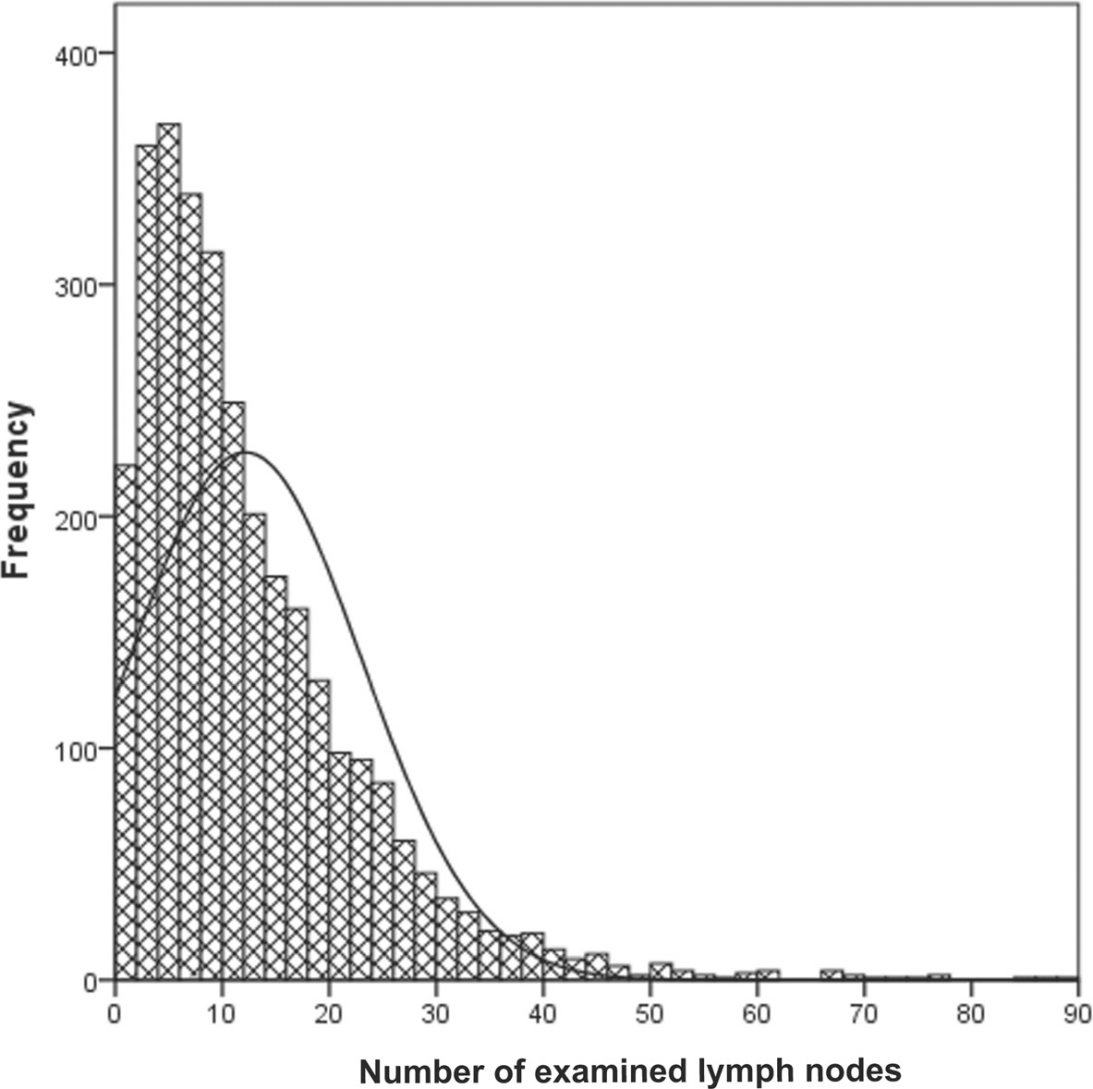
The frequency distribution of examined lymph nodes for the entire cohort of patients

**Fig. 2:**
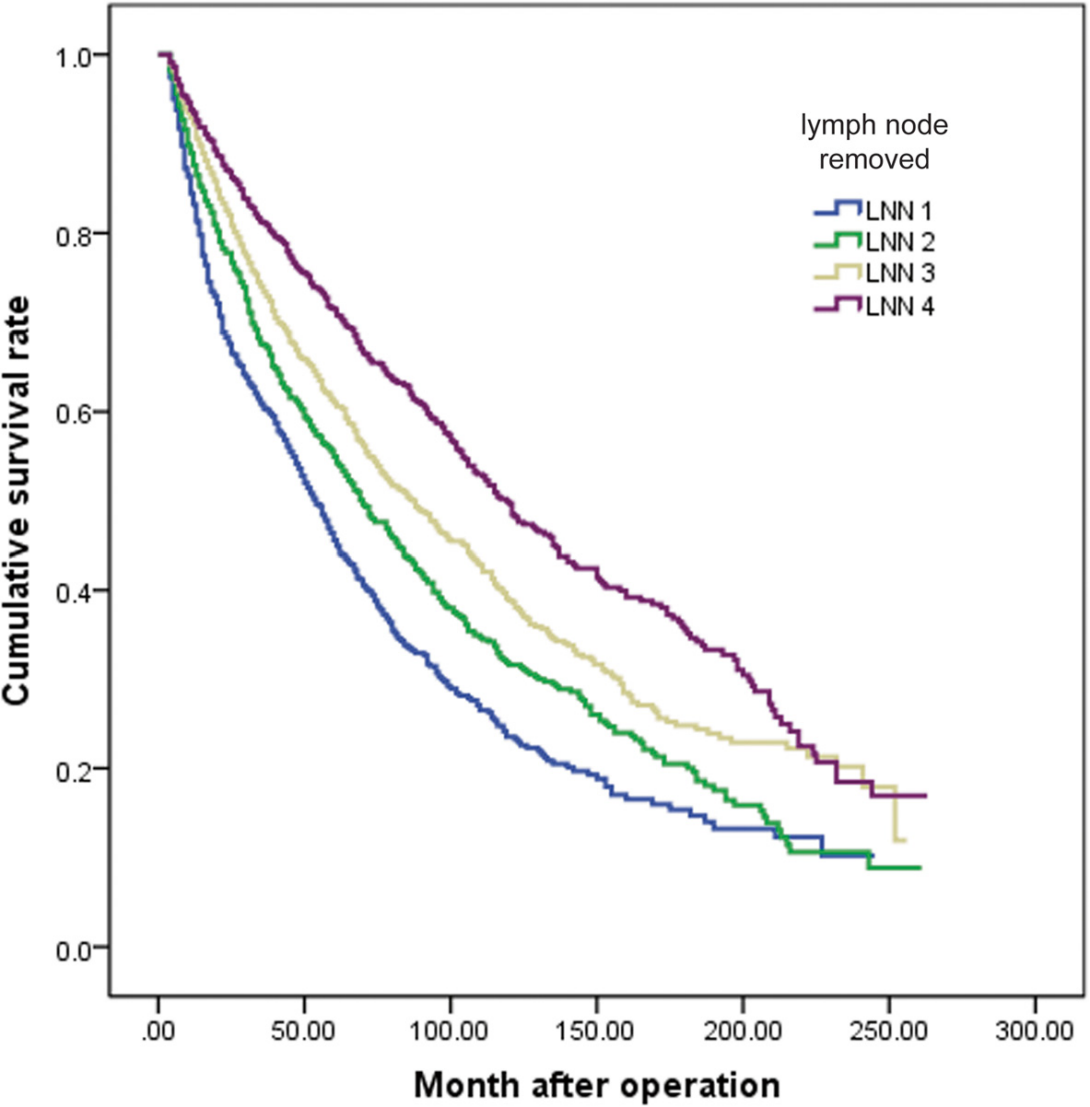
The overall survival curves of different LNN groups

The frequency distribution of examined lymph nodes for the entire cohort of patients is shown in Fig. 1. The overall survival rate of different LNN groups is shown in Fig. 2. The overall survival rate was significantly higher with an increasing number of negative lymph node resected. The 5-year survival rate was 46 % for LNN 1 (patients with 1 to 3 negative lymph nodes removed) compared with the rate of 56, 62, and 72 % for LNN 2, LNN 3, LNN 4, respectively, those who had 4 to 7, 7 to 15 and more than 15 lymph nodes resected (*P* < 0.001).

We used Kaplan-Meier survival analysis to assess seven prognostic factors: age, gender, race, tumor location, tumor size, histopathological grade, and LNN group. Univariate analysis and subsequent multivariate analysis indicated that age, race, tumor size, and LNN group were significant and independent prognostic factors after curative resection for lymph node-negative gastric cancer (Table 2). Next, we extracted the data after 2004 in order to assess the factor of the depth of tumor invasion. At this time, 843 patients were selected and the result of the multivariate analysis confirmed that age, depth of tumor invasion, and LNN group were significant and independent prognostic factors (Table 3).

**Table 2:** Univariate and multivariate predictors of overall survival

	Univariate analysis	Multivariate analysis	
	*P* value	*P* value	Hazard ratio
Age (years)	<0.001	<0.001	2.015 (1.789–2.270)
≤65			
>65			
Gender	0.072		
Male			
Female			
Race	<0.001	<0.001	0.911 (0.878–0.945)
White			
Black			
Indigenous Westerner			
East Asian			
Others			
Location	0.532		
Upper			
Middle			
Lower			
Overlapping			
Tumor size (cm)	<0.001	<0.001	1.006 (1.004–1.008)
Diameter ≤1			
1< Diameter ≤2			
2< Diameter ≤3			
3< Diameter ≤4			
4< Diameter ≤5			
Diameter >5			
Grade	0.066		
Well differentiated			
Moderately differentiated			
Poorly differentiated			
Undifferentiation			
Lymph node-negative group	<0.001	<0.001	0.812 (0.777–0.849)
LNN 1			
LNN 2			
LNN 3			
LNN 4

**Table 3:** Univariate and multivariate predictors of overall survival (data late than 2004)

	Univariate analysis	Multivariate analysis	
	*P* value	*P* value	Hazard ratio
Age (years)	<0.001	<0.001	2.194 (1.529–3.148)
≤65			
>65			
Gender	0.404		
Male			
Female			
Race	0.01	0.724	0.981 (0.881–1.092)
White			
Black			
Indigenous Westerner			
East Asian			
Others			
Location	0.74		
Upper			
Middle			
Lower			
Overlapping			
Tumor size (cm)	<0.001	0.896	1.000 (0.994–1.007)
Diameter ≤1			
1< Diameter ≤2			
2< Diameter ≤3			
3< Diameter ≤4			
4< Diameter ≤5			
Diameter >5			
Depth of invasion	<0.001	<0.001	1.684 (1.463–1.937)
T0 and T1			
T2			
T3			
T4			
Grade	0.654		
Well differentiated			
Moderately differentiated			
Poorly differentiated			
Undifferentiation			
Lymph node-negative group	0.006	<0.001	0.773 (0.674–0.886)
LNN 1			
LNN 2			
LNN 3			
LNN 4			

Only those patients who are diagnosed late than 2004 in the database can get enough details to make an accurate T stage according to the AJCC 7th edition

**Fig. 3:**
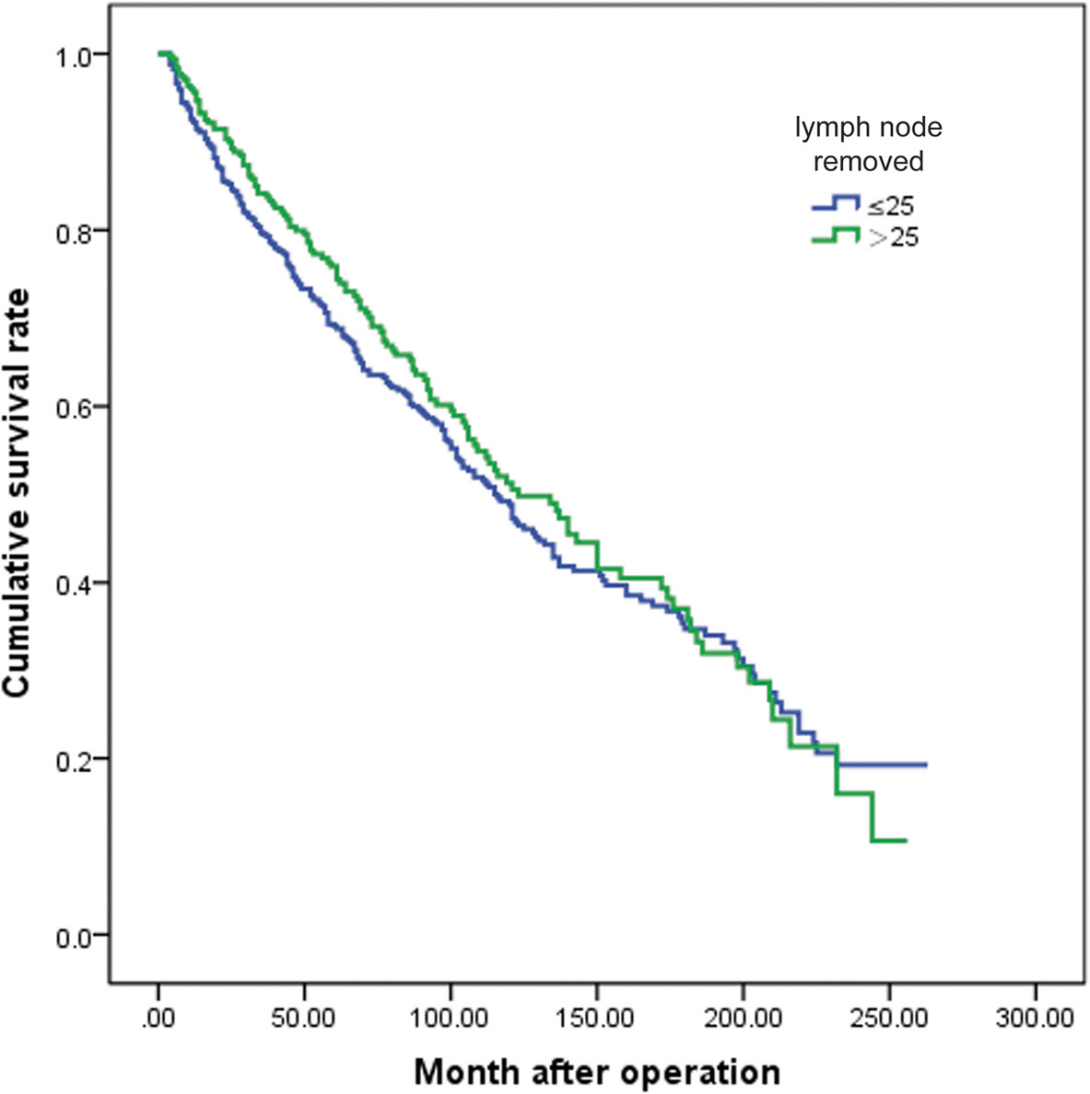
The overall survival curves of patients with more than 25 lymph nodes resected and patients with 15 to 25 lymph nodes resected (log-rank test, *P* = 0.345)

Furthermore, we collected patients with more than 15 lymph nodes removed for the sake of estimation of the value of a more radical lymphadenectomy. Eight hundred seventy-five patients were included according to this criterion. According to the log-rank test result, it did not show a significantly improved survival as a continued lymph nodes resection. A cutoff of 25 lymph node resection was chosen for the analysis on the basis of actual suggestions for the right number of nodes retrieved by a correct D2 dissection [11]. Figure 3 demonstrated that the overall survival rate did not show a significant difference between the patients with more than 25 lymph nodes resected and patients with 15 to 25 lymph nodes resected (*P* = 0.345).

**Fig. 4:**
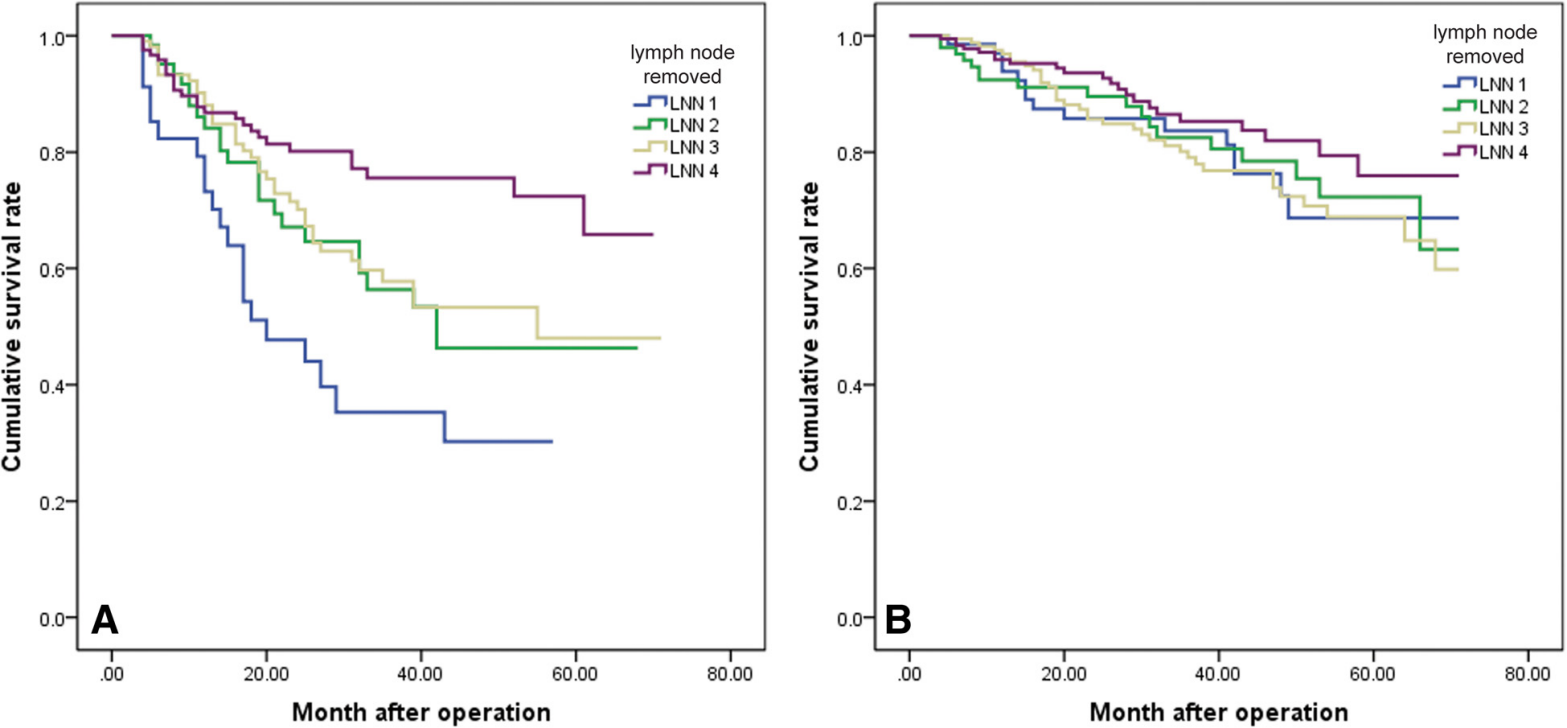
**a** The overall survival rate in patients with T3 + T4 tumors was significantly different between LNN subgroups. **b** The overall survival rate in the patients with T1 + T2 tumors had no statistical difference

Subgroup analysis was then conducted to evaluate the survival of the patients in different pathologic T categories (T1 + T2 and T3 + T4). For patients with T3 + T4 tumors, the overall survival rate was significantly different between LNN subgroups (Fig. 4a). The overall survival rate in patients with T1 + T2 tumors had no statistical difference (Fig. 4b). At the same time, evaluation of the overall survival rate in different tumor size categories indicated that the survival rate was significantly better for patients with more than 15 lymph nodes resected in all the tumor size subgroups.

## Discussion

Our study was based upon rigorous analyses of the previous studies about lymph node-negative gastric cancer patients, thus excluding patients who died within 3 months after operation, patients who had an esophagogastric or cardia cancer (site code C160), patients who had distant metastasis, or patients who had no lymph node resected. The reasons for this criteria setup are as follows: (1) patients died within 3 months likely because of surgical complications; (2) patients who had adenocarcinomas of esophagogastric junction were staged as the esophageal scheme according to the AJCC seventh edition; (3) if the gastric cancer had distant metastasis, it would be a stage IV cancer regardless of the depth and lymph node invasion; and (4) if a patient did not receive the lymphadenectomy, it would be difficult to determine whether the patient had nodal metastases.

Prognostic indicators for high recurrence rate at univariate analysis were ages that were greater than 65 years, the white race, large tumors, deep depth of invasion, and little lymph nodes removed. The association between age and prognosis was hypothesized that elder patients suffer from host immunodeficiency or malnutrition [11, 12]. In our study, tumor location is not a prognostic indicator for lymph node-negative gastric cancer. But this factor is still in controversial. Xu et al. [9] and Baiocchi et al. [11] reported that distal tumors were associated with better prognosis due to a relatively late presentation of the symptoms in proximal gastric cancer [13]. Nevertheless, Lee et al. [14] did not find this phenomenon. Although we divided the tumor location into four groups: upper third, middle third, lower third, and overlapping, we did not find its value to investigate the prognosis. We further combined them into two subtypes: distal gastric cancer and others, and we did not find prognostic value either.

The SEER database is consisted of several registries with different races. Our research indicated that in the univariate analysis, race is a prognostic factor of lymph node-negative gastric patients. The overall survival rate of East Asian patients is better than others. This may be due to the fact that East Asian surgeons routinely perform the D2 lymph node dissection while many Western surgeons perform D1 lymph node dissection [15]. This is supported by the number of lymph nodes resected, combined with the fact that although all the patients underwent D1 or D2 lymph node dissection, the pathologists and surgeons searched for lymph nodes in different efforts and techniques, which could lead to omitted lymph nodes in the specimen [16]. We can see from the results that 40.5 % of East Asian patients received the lymphadenectomy with more than 15 lymph nodes removed, while only 24.2 % of the patients in the Western world received the same type lymphadenectomy.

Tumor size failed to demonstrate a significant association with overall rate in our series in the multivariate analysis. However, the value of these prognostic factors is still in controversial. Baiocchi et al. [11] and Lee et al. [14] found that tumor size (cutoff 40 and 63 mm, respectively) could not independently affect the overall survival. By contrast, Xu et al. [7] indicated that tumor size (cutoff 50 mm) was a strong prognostic factor affecting the survival of lymph node-negative gastric cancer patients. But in this study, tumor size range is analyzed to homogenize the difference by choosing multiple cutoff points which is distinct from other studies using only one cutoff point.

LNN groups, depth of tumor invasion, and ages are independent prognostic indicators for lymph node-negative gastric cancer. The four cutoff points for LNN subgroups indicate that the more lymph nodes resected, the better prognosis the patients can have. After extracting the patients who received the lymphadenectomy with more than 15 lymph nodes removed, however, we found that a more extensive lymphadenectomy (more than 25 lymph nodes resected) did not lead to better survival. For different tumor size subgroups, the overall survival rate among them has statistically significant difference when different lymph nodes were resected, even in the less-than-1-cm group. This means that we cannot judge the extent of lymph node dissection by the tumor size. But for the different depth of invasive subgroups, our study indicated that the overall survival rate for T1 + T2 stage lymph node-negative gastric cancers has no statistically significant difference, while it is statistically significant for the T3 + T4 subgroup. An alternative explanation for this observation may be that patients with T1 or T2 subcategories seldom spread to regional lymph nodes. The incidence of lymph node metastasis in T1 and T2 patients are less than 35 % [17–19]. However, it is difficult to determine the depth of invasion and lymph node metastasis before surgery. Recent studies demonstrated that endoscopic ultrasonography has improved the local accuracy in estimating the depth of tumor invasion and lymph node involvement [20]. The accuracy of endoscopic ultrasonography is better than that of CT scan in determining the extent of infiltration of the tumor. The accuracy ranges from 67 to 92 % [21]. Although the development of technology improves the accuracy in determining the extent of infiltration of gastric cancer, difficulty still remains in differentiating the T2 stage from T3 stage [22]. A high-frequency (up to 30 MHz) miniprobe-endoscopic ultrasonography, which is able to demonstrate gastric wall up to nine different layers, can reach 100 % accuracy in identifying the T1 gastric cancer. But the accuracy of endoscopic ultrasonography is highly dependent on the experience of the operators [22]. Therefore, we strongly suggest that at least 15 lymph nodes be resected during the radical operation.

## Conclusions

In conclusion, although the lymph node-negative gastric cancer has an excellent prognosis, some patients may still have recurrence and die. Age, the number of lymph nodes resected, and the depth of tumor invasion are the prognostic factors to identify the lymph node-negative patients who may receive significant benefit. Further treatments should refer to these indicators. In addition, our study suggests that a lymphadenectomy with more than 15 lymph nodes removed should be performed. But the survival benefit of a lymphadenectomy with 25 and more lymph nodes resected is disputed, and it may need some more evidence to prove its statistically significant survival improvement.
